# Supplementary material for: Genes with epigenetic alterations in human pancreatic islets impact mitochondrial function, insulin secretion, and type 2 diabetes
Source: Nat Commun. 2023 Dec 12;14:8040. doi: 10.1038/s41467-023-43719-9 (PMC10716521; doi:10.1038/s41467-023-43719-9)

Genes with epigenetic alterations in human pancreatic islets impact mitochondrial function, insulin secretion, and type 2 diabetes

Supplementary Information

Supplementary Methods. Pyrosequencing assays used for technical validation of EPIC array data from islets of T2D cases vs. controls (**a-d**) and fine-mapping of *RHOT1* DNA methylation (**e**), as well as the *RHOT1* promoter region for insertion to CpG-free luciferase reporter vector (pCpGL-basic) (**f**).

a. *CDKN1A* cg24425727 chr6:36645648 (hg19)

Forward PCR primer: GGAGTTATAGAAATAAAGGATGATAAGTAG

Reverse PCR primer (biotinylated): TCCCTATAATTACAACAACTTTATTAACCA

Sequencing primer: GAGTTTTAGTTTTTTTAGTAGTG

Sequence to analyze:
TATAYGGGTTATGTGGGGAGTATTTAGGAGATAGATAATTTATTYGTTAAAT
TTTTTTTTTTTGGTTAAT

b. *HDAC4* cg26767974 chr2:240143979 (hg19)

Forward PCR primer: GTATTTGGAGGGAGTAAGATTATTTG

Reverse PCR primer (biotinylated): ACCAAATTTTTATTTTCAACTCTTACTCAA

Sequencing primer: GGAGTAAGATTATTTGTGTG

Sequence to analyze:
TTTTTGTTTTATAATTTAATGAAAAYGTAAGTGTTAGATTTTTTTTTTGTTTT

c. *TXNIP* cg02988288 chr1:145440445 (hg19)

Forward PCR primer: ATGGTTAGATTAAGGTGTTGATTTAGAA

Reverse PCR primer (biotinylated): AATTTCTCTACTAAAACCACCCTACAT

Sequencing primer: GAGGTAATTATATTATTTTAGGGAT

Sequence to analyze:
ATGYGTATTATGGYGTGGTAAGAGTTTTYGGGTTTAGAAGATTAGGTTTTTTATTT

d. *RHOT1* cg10339923 chr17:32206104 (hg38)

Forward PCR primer: AGATGTGTATTATGGGTAGTTGATT

Reverse PCR primer (biotinylated): CAAACCCTCTACATACATTATTTACATTCT

Sequencing primer: ATTATGGGTAGTTGATTTAAT

Sequence to analyze: TTTTTTAGTTYGTTTTTTTATTTGTAGAATGTAAAT

e. *RHOT1* chr17:32206263-32206266 (hg38)

Forward PCR primer: TTTATTTTTGTTATTTAGGTTGGAATGT

Reverse PCR primer (biotinylated): AATTCTATAAAAACTTAACCAAATACAA

Sequencing primer: TGTTATTTAGGTTGGAATGTA

Sequence to analyze:
GYGGYGTGATTTYGGTTTATTGTAATTTTTGTTTTTTAGGTTTAAGYGATTTTTTT
GTTTTAGTTTTTTTAGTAGTTGGGATTATAGGTATAYGTTATTGTATTTGGTTAAGTTTTTA

f. *RHOT1* promoter region for insertion to CpG-free luciferase reporter vector (pCpGL-basic).

hg38 chr17:32,141,141-32,142,680

AGATTGCGCCACTGCACTCCAGCCTGGGCGACAGAGCGAGACTCCGTCTCAAAACAAAACCAAAACAAAACAA
AAAATCTGTGGATTACAACTTTTTAGTGACTTTTTAATATAATAATGATAGCTGAGATCAGTAGAGCACACGCT
CTGAGTCTGGCACTGTACCAAATGCTTGACAAGTATTATAGTATTTATTACTCACAACCATCTCAAGAGGAAGG
TATTATCATCATTATTTCCATGCTACAGAAGAAGGAACAGTGGCTGAGGGAAGTAAAAATAAAAGCACTTAGT
CGAGATCACAGAGCTATTATGTGGAGCCTAGATTCAAACCCAGGAAATTTGCCAGCAACTCTTAACTGCTGCAA
GAGACCACATGACTTCTCCTAGGAGAAAGGGAAGTTGGTATTACGCACATTTCATTCCCTTTAAGTGTTTATTG
TGTAATATTTAAGATAGAAAAAATGTACATTATGCACAGATAGTGCTTCCTCATTAATTTATGTATTTTATATTTC
CCACTGTGTGCCAAGTATTAGGGATGTGATATATATTCGCACGCGTATGTATATATATATATTTGCCATTGTGTA
CCAAACATTAAGCATGTAACAACAAATTCCTCCAGATGGAGAGGGTCACGAGAAAATTAAGGCAATGTTAGAA
AAATCATCTAGAGTTTATTATCGAGTGCCACCTCCGTGTCTAGCATTACGCTGGGTAAGCCTGAGTAAAGAAGA
GTGTCCGAAAACCCTGCCATAAAATAAACTACATAGACAGAAAAAAAGTCACTTAGTTTGGTTATAAAGAAAG
GAGTCTTGTTCACACAGCGTAAAAACGAATCTTATAATCGTATTTTATAAGCATCAGAGGCAAAAGCAACTAGG
GTGACGGGCTTTGGGGAGTGGAAAGCGCCGGATCCTGGGAGCCAGTCGCCCTACTCGATCAAGCATTTTCTG
ACGGCCCTATCCAATAACGTGGGGACGCAGCTGAAAACCATGCAAGCTGATCGCTGCCCTCCCGGAGTTTATG
GTCTGGTGAGAAGATCCCTGCTCTGAGTCACTGCCAAGCTCTAGCTATGTGACTTTGAACAGGGAATTTCTGTC
CCTATCTATAAAATAAACGGGGTTAAACCAGGGAATCCGCCATTCAAATTCCTGGCCCAAGTGTCTCCGGGGTC
GCGGTGCCCAAAGGAGGGTGGGCGAGGCTCCCTGGGCCTGCGCTGCGGCGGCCTGACAGCGGGAGCGCTGT
TCCGGCGCAGAGCGCGTTCGCGTTCCGAAAGCGCGTGTCCCTCGCCGCGTGCCGTCGTCCCCGCCGCCGCC
CGCCGCCGCCGCCGCCGCCGCCGCCGCCGCCGCCGCCACAGCCCGCTGGGCCGGAGGAGGCGGAGCTGGCG
CTGTCCCGGCTCTCTTGCGGGGAAGCAACTGAGGGGGCGGCGCGGCGGGCCCCGGCGGCCGAAGAGGCTGG
CAGGTGGCGCCGTGGGGTGGGTGCTCCTGGTGAGAGGAGTCCACTCCGTGCGTGCGGGCGGAGGCCGGCCC
CCG

Supplementary Figures

Supplementary Fig. 1. The islet EPIC array methylation study validated data from previous islet methylome studies. Differential DNA methylation identified in the *Islet T2D case-control cohort* confirmed results from our previous human islet methylome studies[1-2]. DNA methylation analysis in the current *Islet T2D case-control cohort* found altered methylation of several sites annotated to *CACNA1C* (**a**), *CDKN1A* (**b**), *GLP1R* (**c**), *HDAC4* (**d**), *HDAC7* (**e**), *IL6R* (**f**), *KCNQ1* (**g**), *PDE7B* (**h**), *THADA* (**i**), *DNMT3A* (**j**), *PDX1* (**k**), *TCF7L2* (**l**), and *WFS1* (**m**) in islets from 25 T2D cases vs. 75 controls. Data shown as mean ± SEM, *q<0.05 for T2D cases vs. controls. Source data are provided as a Source Data file.

Supplementary Fig. 2. Differential DNA methylation identified in the *Islet T2D case-control cohort* overlap open chromatin regions (OCRs) in pancreatic islets from T2D cases vs. controls[3]. a,b) *SLC7A2* (**a**) and *WFS1* (**b**) have more prevalent ATAC-seq peaks, representing OCRs, and lower levels of DNA methylation in islets from 25 T2D cases vs. 75 non-diabetic controls. **c**) An OCR covering *PDE7B* TSS has eight sites with lower DNA methylation in islets from 25 T2D cases vs. 75 controls. Data shown as mean ± SEM, *q<0.05 for T2D cases vs. controls. Source data are provided as a Source Data file.

Supplementary Fig. 3. Most genes differentially expressed in islets from T2D cases also had differential DNA methylation in the *Islet T2D case-control cohort*. a-e) Inverse relationships between DNA methylation and gene expression were observed for *CDKN1C* (**a**), *GAD1* (**b**), *GLRA1* (**c**), *IL6* (**d**), and *RBP4* (**e**) in islets from 25 T2D cases vs. 75 controls. Some genes with both differential DNA methylation and gene expression in islets from T2D cases vs. controls also overlap with islet-specific markers of transcriptional regulation[4-5], open chromatin regions (OCRs)[3], and whole-genome bisulfite sequencing (WGBS) of human islets[2]. **f**) Increased DNA methylation of three sites and decreased expression of *SLC2A2* in islets from T2D cases overlap markers of an active enhancer and binding regions for two transcription factors (PDX1 and NKX6.1). Increased methylation in islets from T2D cases was also observed for *SLC2A2* using WGBS[2]. **g**) Relative differences of promoter DNA methylation and gene expression in islets of T2D cases versus controls. Genes were selected based on differential expression in islets from T2D donors, and one or more differentially methylated CpG site(s) annotated to the promoter region (TSS200, TSS15000) of the same gene. **h**) *BEST3* has 50% decreased expression in islets from T2D cases vs. controls. In an active promoter region around the TSS known to be bound by NKX2.2, MAFB, and PDX1, there are three sites with increased DNA methylation in islets from 25 T2D cases vs. 75 controls. This region also resides within an OCR. Chromosomal positions are based on hg38. Data shown as mean ± SEM, *q<0.05 for T2D cases vs. controls. Source data are provided as a Source Data file.

Supplementary Fig. 4. Weighted combined methylation risk scores (MRSs) for genes with altered expression and more than five differentially methylated CpG sites within or near (±10 kb) that gene. a-f) MRSs with difference between T2D cases vs. controls in multiple linear regression models, displaying the combined effect of several differentially methylated sites on T2D in human islets. Box plots indicate median (middle line), 25 – 75 percentile (box) and 5 - 95 percentile (whiskers) as well as outliers (single points). **g)** Higher values of MRSs in human islets were associated with a higher risk of having T2D (OR per 1% increase in methylation). Source data are provided as a Source Data file.

Supplementary Fig. 5. Functional validation in human pancreatic islets and INS-1 832/13 β-cells. a) No differences were observed in insulin content in human islets silenced for *FOXP1*, *TBC1D4*, *RHOT1,* or *CABLES1* vs. siNC at 2.8 mM or 16.7 mM glucose. Data are presented as mean ± SEM, *n*=5 independent experiments in different islet donors. p>0.05 vs. siNC based on a paired t-test. **b**) Global gene expression based on microarray analysis in human pancreatic islets silenced for *FOXP1*, *TBC1D4*, *RHOT1,* or *CABLES1* using siRNA, confirming a significant knockdown of these four candidate genes compared with siNC. Data are presented as mean ± SEM, *n*=5 independent experiments in different islet donors, *p<0.05 vs. siNC based on a paired t-test. **c,d**) Correlations between *RHOT1* and *LAMP2* (**c**) or *MFN2* (**d**) expression based on RNA-seq data for pancreatic islets from 109 non-diabetic donors. Pearson correlation coefficient *r* is presented with fitted lines based on linear regression and 95% confidence interval. **e**) Transcription factors (TFs) binding to the *RHOT1* promoter and being differentially expressed after silencing of *FOXP1*, *CABLES1* or *TBC1D4*. 619 binding sites were identified 0-1000 bp upstream of *RHOT1*[6], of which 614 overlapped regions defined as promoters based on ATAC-Seq and histone modifications in human islets[5]. Among them, 7, 8 and 13 were differentially expressed after silencing of *FOXP1*, *CABLES1* or *TBC1D4*, respectively. The Venn diagram shows that one TF with the ability to bind in the *RHOT1* promoter of human islets were affected by all siRNAs. **f**) Luciferase assay measuring the transcriptional activity of the *RHOT1* promoter region without methylation (Ctrl) and after in vitro methylation using SssI (90 CG sites). Individual values normalized to renilla are shown, and mean ± SEM. **p<0.005 based on a paired t-test, *n*=4. **g**) Insulin secretion normalized to insulin content in *Rhot1*-deficient β-cells. Data are presented as mean ± SEM, *n*=6 independent experiments. $^{\#}$p<0.05 at 2.8 mM and *p<0.05 at 16.7 mM glucose for siRhot1 vs. siNC based on a Wilcoxon matched-pairs signed rank test. **h**) Western blot showing Rhot1 protein levels in *Rhot1*-deficient β-cells vs. control (siRhot1 vs. siNC; *n*=4 independent experiments) with target signal normalized to the total amount of protein in each lane. **i**) Total protein signal used for normalization of Rhot1 protein expression using ImageLab software version 6.0.1 (V3 Western Workflow, Bio-Rad). siNC: non-target control. Source data are provided as a Source Data file.

Supplementary Fig. 6. Metabolic characterization of *Rhot1*-deficiency in INS-1 832/13 β-cells. a-c) Reduced protein levels of citrate synthase (**a**), cytochrome C (**b**), and five components of the electron transport chain (**c**) in *Rhot1*-deficient β-cells. Data are based on a paired t-test, *p<0.05 and **p<0.005 for siRhot1 vs. siNC, *n*=6 independent experiments. Target signal is normalized to the total amount of protein in each lane. **d-f**) Analysis of mitochondrial superoxide production in INS-1 823/13 β-cells. Cells were stained with the mitochondrial probe MitoSox Red. **d**) Living INS-1 823/13 β-cells were selected using the forward vs. side scatter (FSC vs. SSC) gating strategy. **e**) Representative mean fluorescent intensity (MFI) of mitochondrial superoxide production by mock-transfected INS-1 β-cells and β-cells transfected with siNC or siRhot1. **f**) Quantified MFI of mitochondrial superoxide production by mock-transfected and transfected INS-1 β-cells. Data are presented as mean ± SEM, *n*=9 independent experiments, **p<0.005 for siRhot1 vs. mock or siNC based on a Wilcoxon matched-pairs signed rank test. siNC: non-target control. Source data are provided as a Source Data file.

Supplementary Fig. 7. Altered levels of autophagy markers in INS-1 832/13 β-cells silenced for *Rhot1* and Rhot1 protein in a rodent model of T2D. a-c) Western blot showing protein levels of Lamp1 (**a**), p62 (**b**), and Lc3b (Lc3b-II is the smaller lipidated isoform) (**c**) under normal (control) and starved conditions (3 h amino acid starvation). Differences between siNC and siRhot1 are based on

paired t-tests, *p<0.05 and **p<0.005 for siRhot1 vs. siNC, *n*=7 independent experiments. Target signal is normalized to the total amount of protein in each lane. **d**) Western blot showing increased levels of the short isoform of Opa1 and a reduced ratio of long vs. short Opa1 isoforms in *Rhot1*-deficient INS-1 832/13 β-cells. Differences between siNC and siRhot1 are based on paired t-tests, *p<0.05 and **p<0.005 for siRhot1 vs. siNC, *n*=12 independent experiments. Target signal is normalized to the total amount of protein in each lane. **e-g**) Western blot showing protein levels of RHOT1 (**e**), CS (**f**), and five components of the electron transport chain (NDUFB8, SDHB, UQCRC2, MTCO1, and ATP5A) (**g**) in adult (12 weeks of age; *n*=4) vs. young (8 weeks of age; *n*=4) GK rats and control Wistar rats (12 weeks of age; *n*=5) (top). Protein levels were normalized to the total amount of protein in each lane (bottom) using ImageLab software version 6.0.1 (V3 Western Workflow, Bio-Rad). siNC: non-target control. Source data are provided as a Source Data file.

Full blots

Fig S6a: Citrate synthase

Si-experiment blot, n = 6.

Total protein blot.

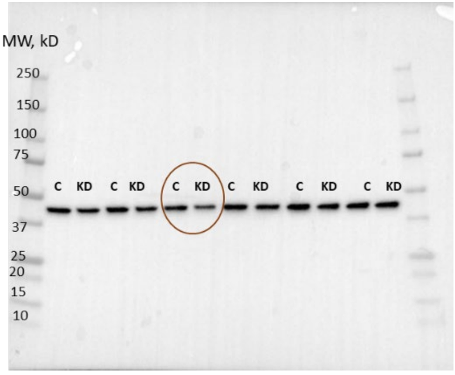

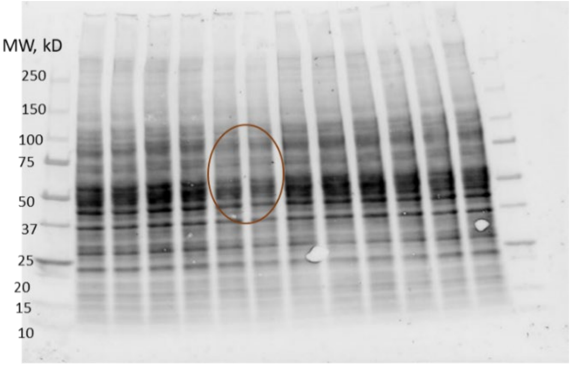

Fig S6b: Cytochrome C

Si-experiment blot, n = 6.

Total protein blot.

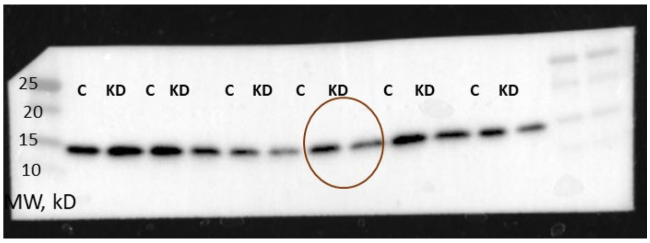

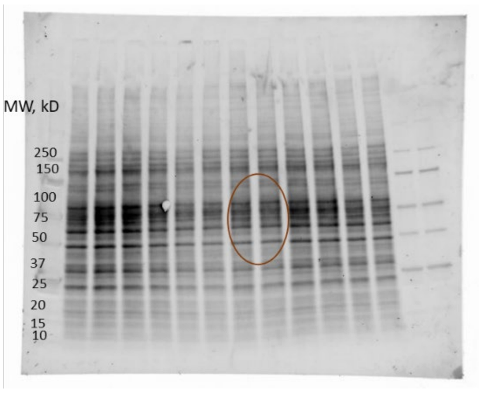

Fig S6c: OXPHOS

Si-experiment blot, n = 6.

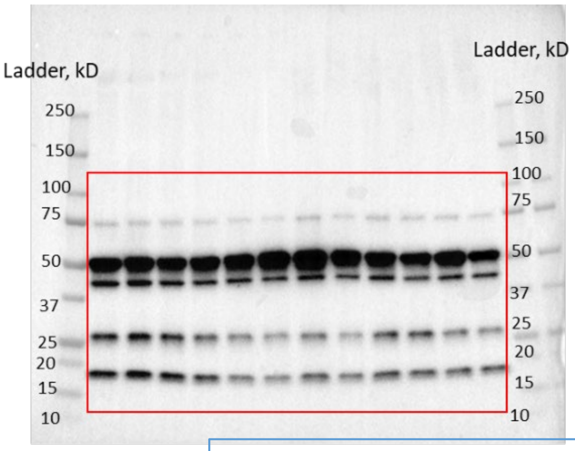

Total protein blot.

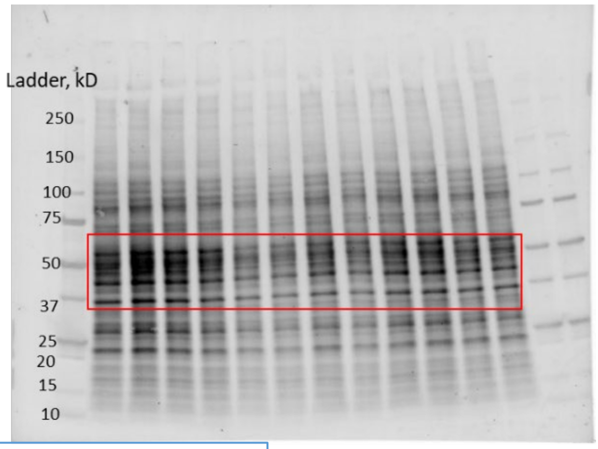

C =siNC KD=siRHOT1, paired t-test

Fig S7a-c: Total protein blot – p62, Lc3b, Lamp1 blots

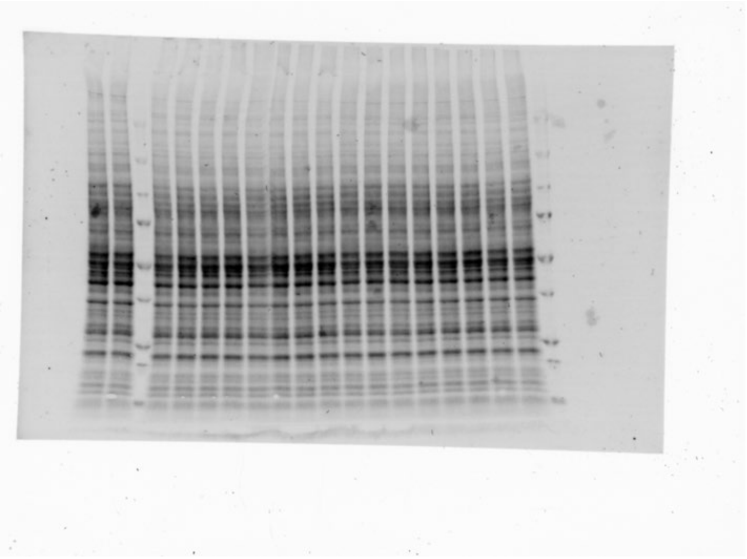

Fig S7d: Total protein blot – Opa1 blot

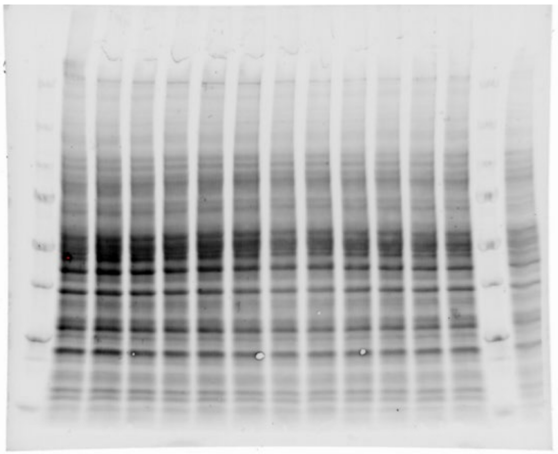

Supplementary References

1. Dayeh, T. *et al.* Genome-wide DNA methylation analysis of human pancreatic islets from type 2 diabetic and non-diabetic donors identifies candidate genes that influence insulin secretion. *PLoS Genet* **10**, e1004160 (2014).
2. Volkov, P. *et al.* Whole-Genome Bisulfite Sequencing of Human Pancreatic Islets Reveals Novel Differentially Methylated Regions in Type 2 Diabetes Pathogenesis. *Diabetes* **66**, 1074-1085 (2017).
3. Bysani, M. *et al.* ATAC-seq reveals alterations in open chromatin in pancreatic islets from subjects with type 2 diabetes. *Scientific Reports* **9** (2019).
4. Pasquali, L. *et al.* Pancreatic islet enhancer clusters enriched in type 2 diabetes risk-associated variants. *Nat Genet* **46**, 136-143 (2014).
5. Miguel-Escalada, I. *et al.* Human pancreatic islet three-dimensional chromatin architecture provides insights into the genetics of type 2 diabetes. *Nat Genet* (2019).
6. Kolmykov, S. *et al.* GTRD: an integrated view of transcription regulation. *Nucleic Acids Res* **49**, D104-D111 (2021).

Supplementary Data

Supplementary Data 1. Sites with differential DNA methylation between pancreatic islets from cases with type 2 diabetes (*n*=25) and non-diabetic controls (*n*=75) (q<0.05).

Supplementary Data 2. KEGG pathway analysis based on genome-wide DNA methylation data for human islets from T2D cases (*n*=25) and non-diabetic controls (*n*=75). Associations with T2D based on FDR-adjusted p-value < 0.05.

Supplementary Data 3. Sites with an association between HbA1c and DNA methylation in human pancreatic islets from 114 donors not previously diagnosed with T2D (the Islet HbA1c cohort, q<0.05).

Supplementary Data 4. Sites with both differential DNA methylation between human pancreatic islets from T2D cases (*n*=25) and non-diabetic controls (*n*=75, q<0.05) and an association between HbA1c and DNA methylation in human pancreatic islets from 114 donors not previously diagnosed with T2D (The Islet HbA1c cohort, q<0.05).

Supplementary Data 5. KEGG pathway analysis based on genome-wide DNA methylation data for human islets from 114 donors not previously diagnosed with T2D (the Islet HbA1c cohort). Associations with HbA1c were based on FDR-adjusted *p*-value < 0.05.

Supplementary Data 6. Clinical characteristics of the 83 donors of pancreatic islets used for pyrosequencing replication.

Supplementary Data 7. Sites with differential DNA methylation between pancreatic islets from T2D cases (*n*=25) and controls (*n*=75) in the present study that were also identified using the previous 450k array in human islets from 15 T2D cases and 34 control donors[4] (Sheet A). Sites with differential DNA methylation between human pancreatic islets from T2D cases (*n*=25) and controls (*n*=75) in previously

identified differentially methylated regions (DMRs) between islets from six T2D cases and eight controls[6] (Sheet B). Sites with an association (q<0.05) between HbA1c and DNA methylation in human pancreatic islets from donors not previously diagnosed with T2D (Islet HbA1c cohort, *n*=114) and also identified in differentially methylated regions (DMRs) between islets from six T2D cases and eight control donors[6] (Sheet C).

Supplementary Data 8. Sites with differential DNA methylation between pancreatic islets from T2D cases (*n*=25) and non-diabetic controls (*n*=75) in islet open chromatin regions as reported by Bysani et al.[16].

Supplementary Data 9. Sites with differential DNA methylation between human pancreatic islets from T2D cases (*n*=25) and non-diabetic controls (*n*=75) within 10 kb of genes that are also differentially expressed based on RNA-seq data for 97 samples from the Islet T2D case-control cohort (q<0.05).

Supplementary Data 10. Weighted combined methylation risk score (MRS) for each gene with altered expression and more than five differentially methylated CpG sites within or near (±10 kb) that gene in the Islet T2D case-control cohort.

Supplementary Data 11. Sites with differential DNA methylation between human pancreatic islets from T2D cases (*n*=25) and controls (*n*=75) in regions bound by islet-specific transcription factors[24].

Supplementary Data 12. Sites with differential DNA methylation between human pancreatic islets from T2D cases (*n*=25) and controls (*n*=75) within islet regulatory elements[26].

Supplementary Data 13. Characteristics of participants in the prospective matched case-control study of EPIC-Potsdam.

Supplementary Data 14. Overlap of T2D-associated genes and specific CpG sites from previously conducted prospective or incident T2D studies in blood and differential methylation in the present ***Islet T2D case-control cohort***.

Supplementary Data 15. Differential gene expression in human islets from non-diabetic donors after silencing by siRNA for *CABLES1*, *FOXP1*, *RHOT1,* or *TBC1D4* (five experiments in *n*=5 different donors per group).

Supplementary Data 16. Transcription factor binding sites in positions -1000 to 0 of *RHOT1*[39] in regions defined as promoters in human islets based on ATAC-Seq and histone modifications[26], and overlap with differentially expressed genes after silencing of *FOXP1*, *CABLES1* or *TBC1D4*.

Supplementary Data 17. Metabolomics of INS-1 β-cells transfected with siRhot1 or siNC (non-template control).

Supplementary Figure 1

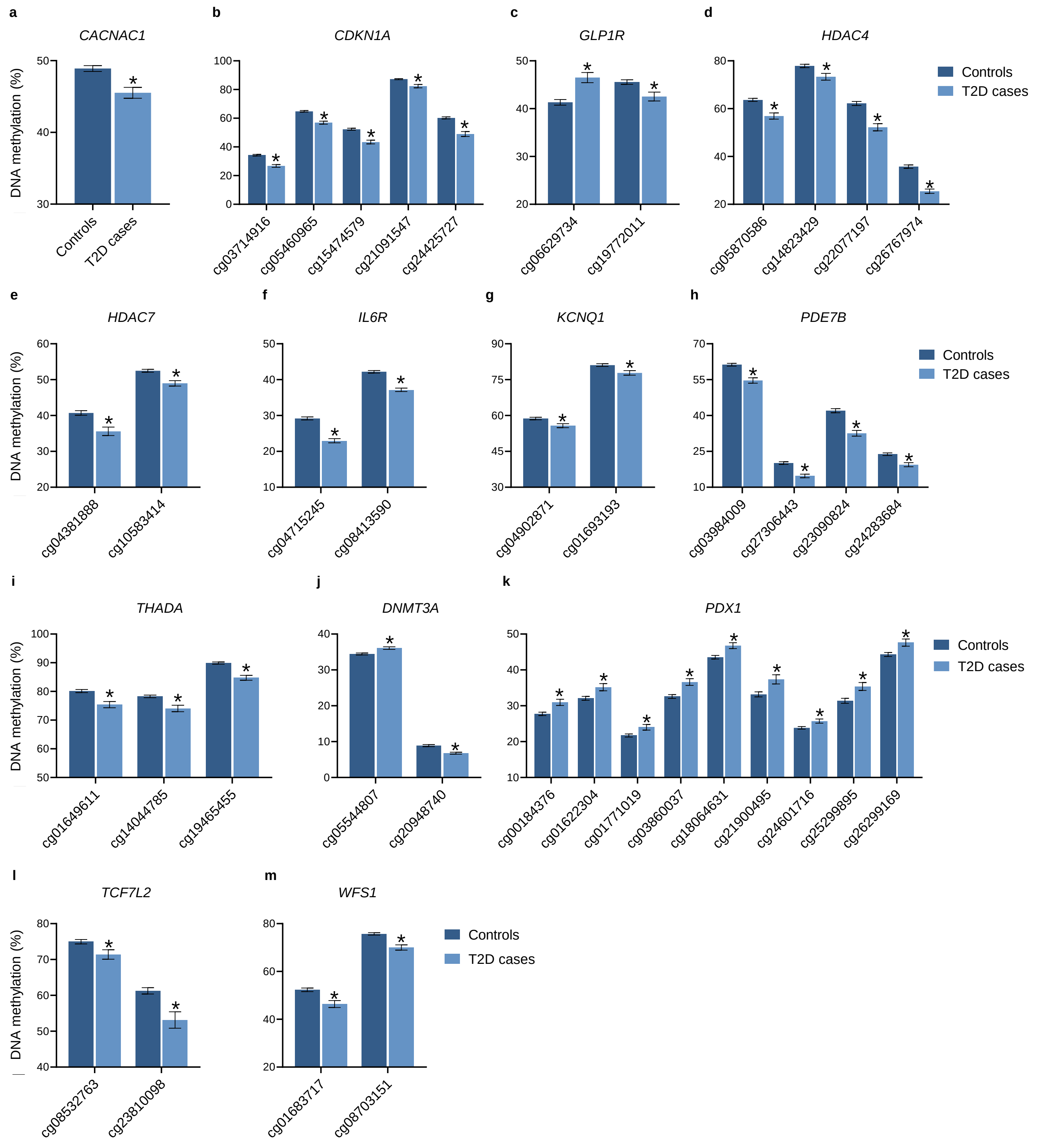

Supplementary Figure 2

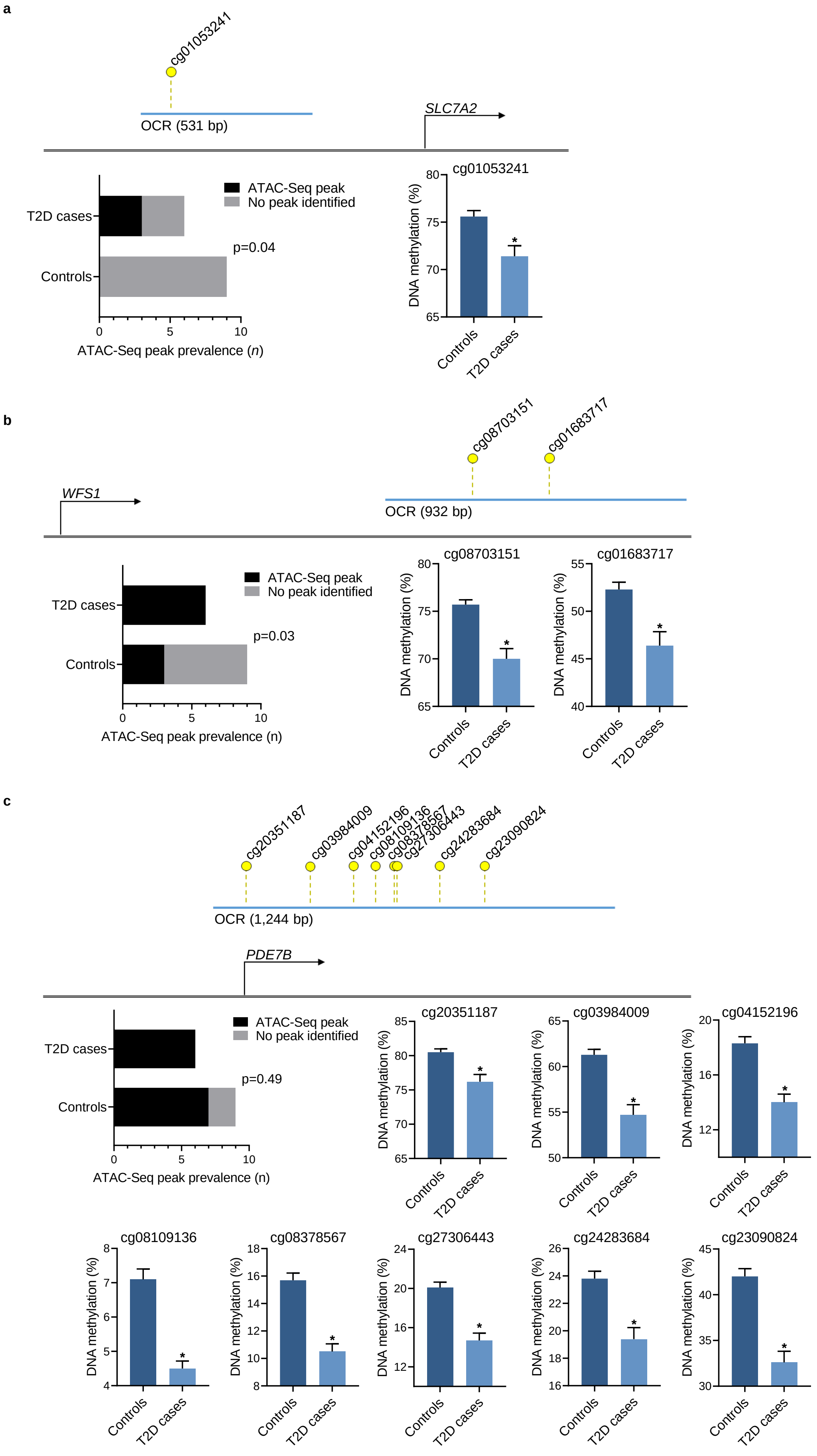

Supplementary Figure 3

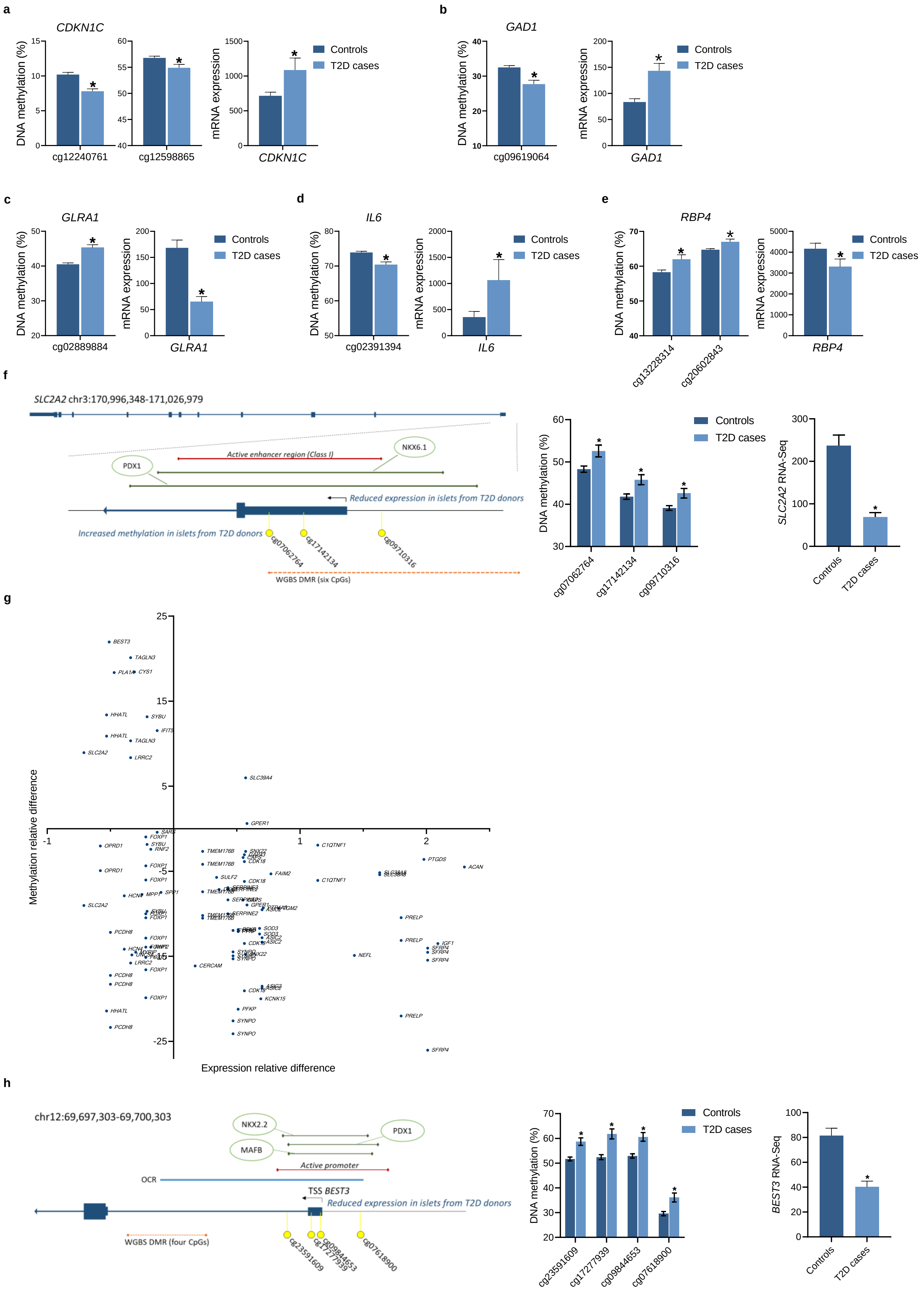

Supplementary Figure 4

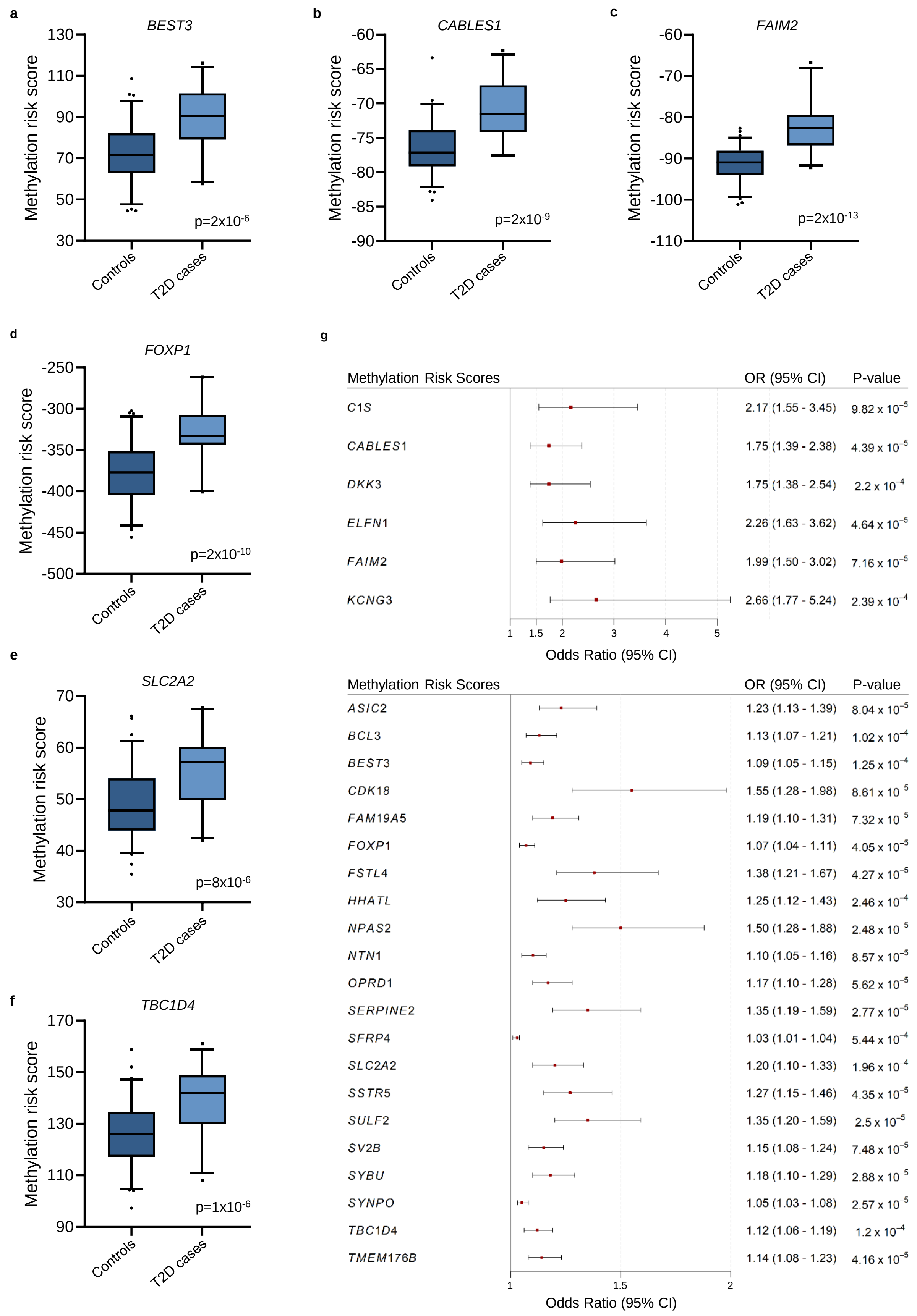

Supplementary Figure 5

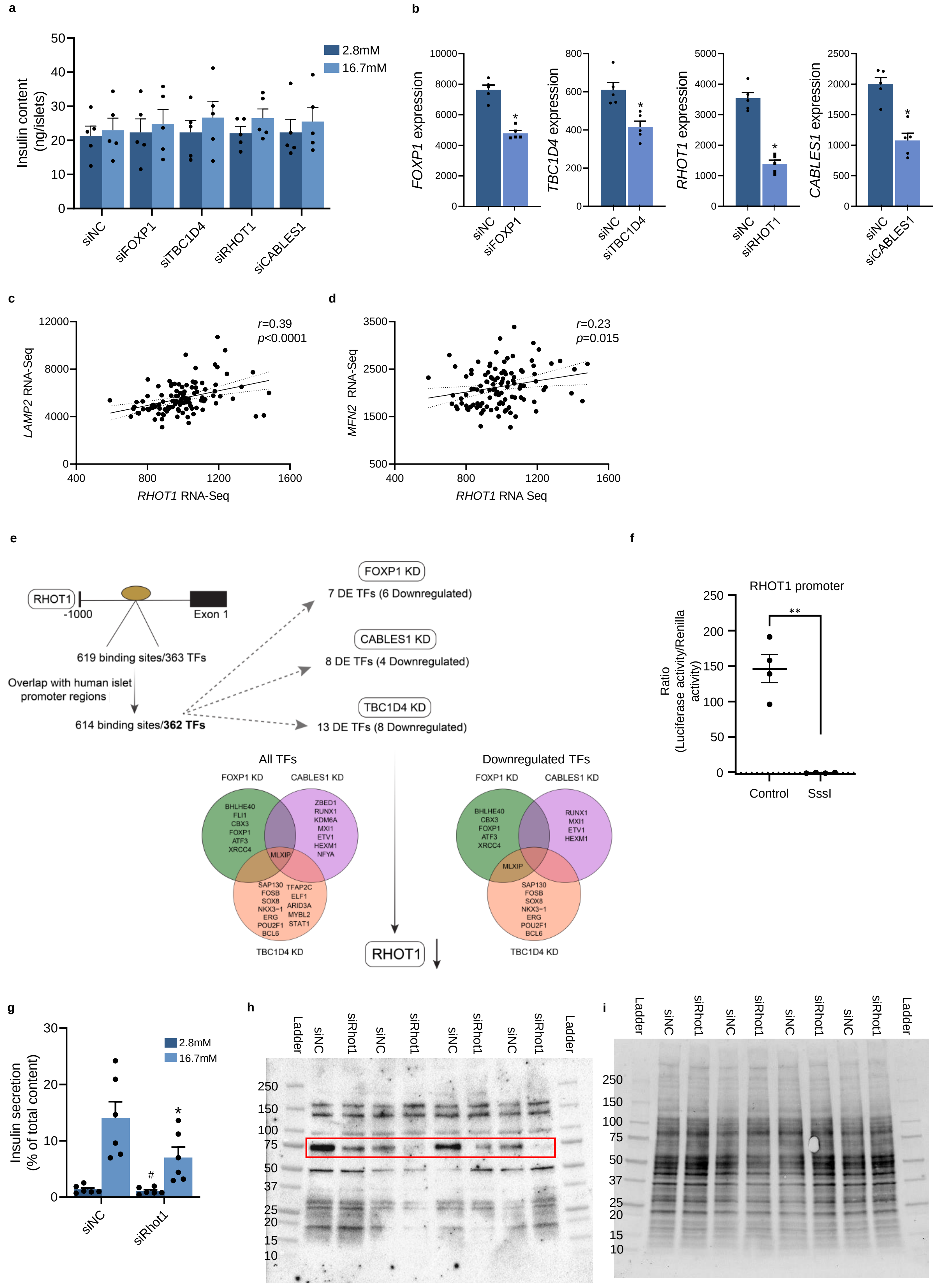

Supplementary Figure 6

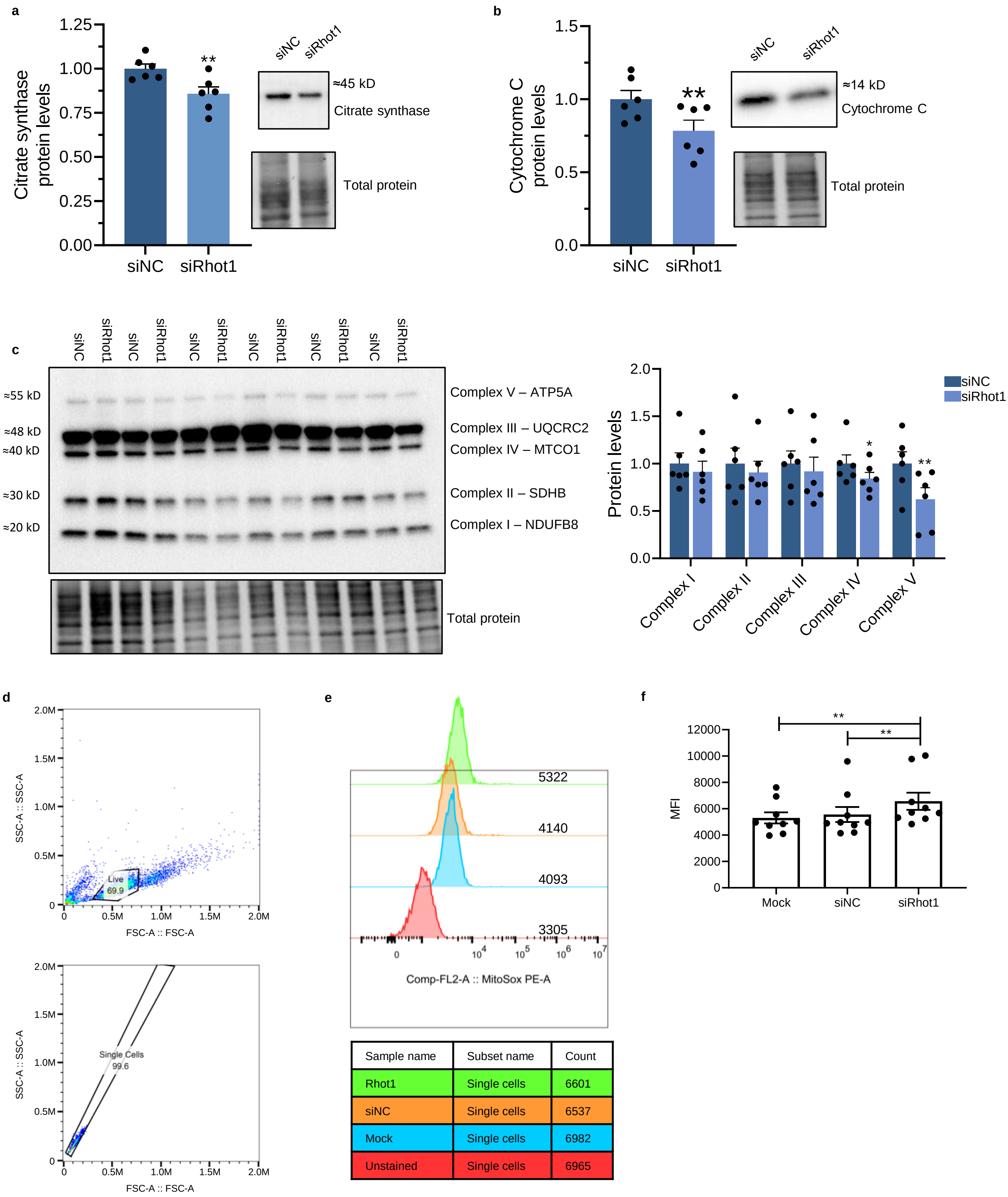

Sample name	Subset name	Count
Rhot1	Single cells	6601
siNC	Single cells	6537
Mock	Single cells	6982
Unstained	Single cells	6965

Supplementary Figure 7

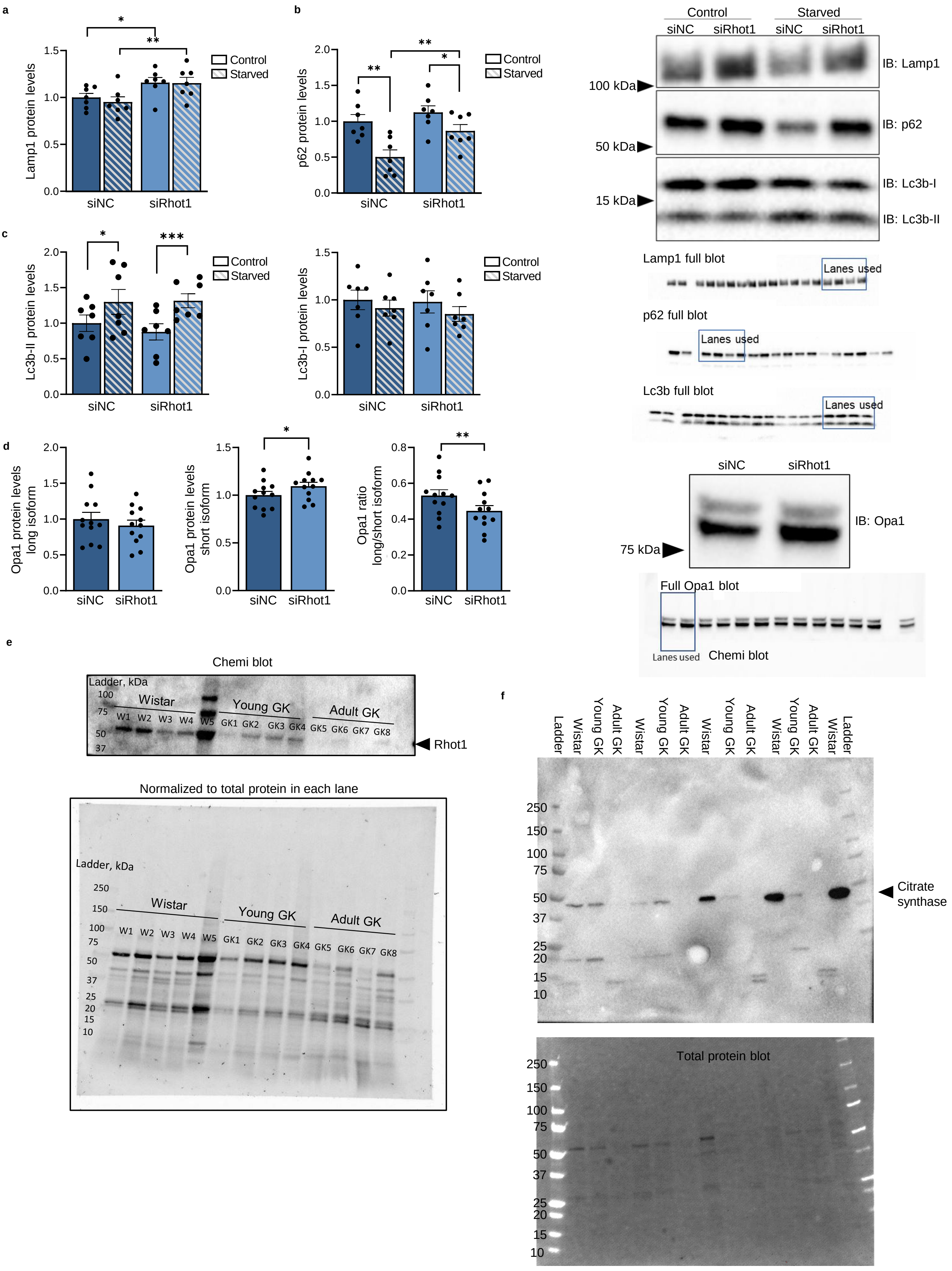

Supplementary Figure 7

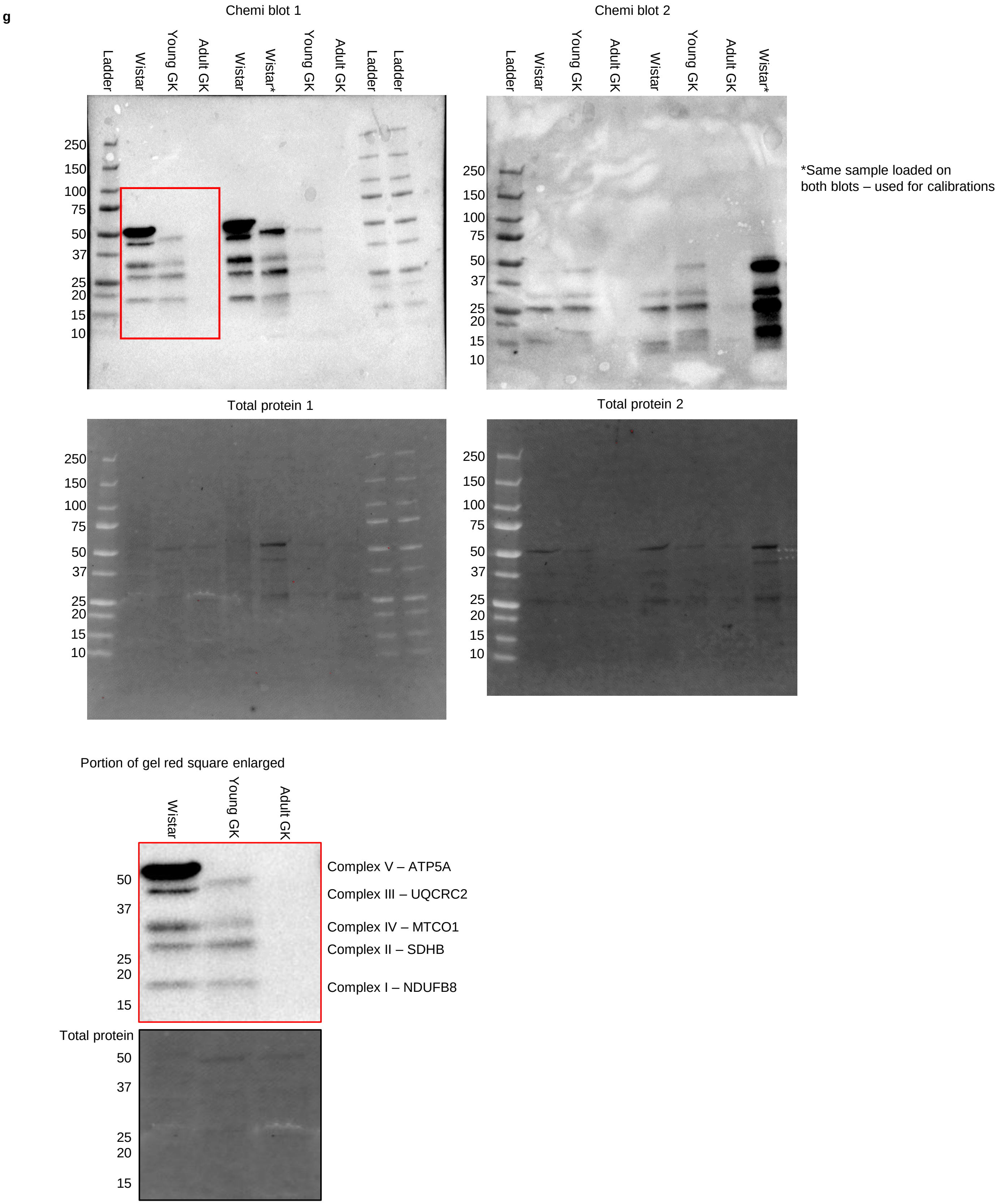

Supplement: Supplementary file 1 — Supplementary Information [file 41467_2023_43719_MOESM1_ESM.pdf]
