# Supplementary material for: Genes with epigenetic alterations in human pancreatic islets impact mitochondrial function, insulin secretion, and type 2 diabetes
Source: Nat Commun. 2023 Dec 12;14:8040. doi: 10.1038/s41467-023-43719-9 (PMC10716521; doi:10.1038/s41467-023-43719-9)
Supplement: Supplementary file 3 — Description of Additional Supplementary Files [file 41467_2023_43719_MOESM3_ESM.pdf]

Description of Additional Supplementary Files

File Name: Supplementary Data 1
Description: Sites with differential DNA methylation between pancreatic islets from cases with type 2 diabetes (*n*=25) and non-diabetic controls (*n*=75) (q<0.05).

File Name: Supplementary Data 2
Description: KEGG pathway analysis based on genome-wide DNA methylation data for human islets from T2D cases (*n*=25) and non-diabetic controls (*n*=75). Associations with T2D based on FDR-adjusted p-value < 0.05.

File Name: Supplementary Data 3
Description: Sites with an association between HbA1c and DNA methylation in human pancreatic islets from 114 donors not previously diagnosed with T2D (the Islet HbA1c cohort, q<0.05).

File Name: Supplementary Data 4
Description: Sites with both differential DNA methylation between human pancreatic islets from T2D cases (*n*=25) and non-diabetic controls (*n*=75, q<0.05) and an association between HbA1c
and DNA methylation in human pancreatic islets from 114 donors not previously diagnosed with T2D
(The Islet HbA1c cohort, q<0.05).

File Name: Supplementary Data 5
Description: KEGG pathway analysis based on genome-wide DNA methylation data for human islets from 114 donors not previously diagnosed with T2D (the Islet HbA1c cohort). Associations with HbA1c were based on FDR-adjusted *p*-value < 0.05.

File Name: Supplementary Data 6
Description: Clinical characteristics of the 83 donors of pancreatic islets used for pyrosequencing replication.

File Name: Supplementary Data 7
Description: Sites with differential DNA methylation between pancreatic islets from T2D cases (*n*=25) and controls (*n*=75) in the present study that were also identified using the previous 450k array in human islets from 15 T2D cases and 34 control donors[4] (Sheet A). Sites with differential DNA methylation between human pancreatic islets from T2D cases (*n*=25) and controls (*n*=75) in previously identified differentially methylated regions (DMRs) between islets from six T2D cases and eight controls[6] (Sheet B). Sites with an association (q<0.05) between HbA1c and DNA methylation in human pancreatic islets from donors not previously diagnosed with T2D (Islet HbA1c cohort, *n*=114) and also identified in differentially methylated regions (DMRs) between islets from six T2D cases and eight control donors[6] (Sheet C).

File Name: Supplementary Data 8
Description: Sites with differential DNA methylation between pancreatic islets from T2D cases (*n*=25) and non-diabetic controls (*n*=75) in islet open chromatin regions as reported by Bysani et al.[16].

File Name: Supplementary Data 9
Description: Sites with differential DNA methylation between human pancreatic islets from T2D cases (*n*=25) and non-diabetic controls (*n*=75) within 10 kb of genes that are also differentially expressed based on RNA-seq data for 97 samples from the Islet T2D case-control cohort (q<0.05).

File Name: Supplementary Data 10
Description: Weighted combined methylation risk score (MRS) for each gene with altered expression and more than five differentially methylated CpG sites within or near (±10 kb) that gene in the Islet T2D case-control cohort.

File Name: Supplementary Data 11
Description: Sites with differential DNA methylation between human pancreatic islets from T2D cases (*n*=25) and controls (*n*=75) in regions bound by islet-specific transcription factors[24].

File Name: Supplementary Data 12
Description: Sites with differential DNA methylation between human pancreatic islets from T2D cases (*n*=25) and controls (*n*=75) within islet regulatory elements[26].

File Name: Supplementary Data 13
Description: Characteristics of participants in the prospective matched case-control study of EPIC-Potsdam.

File Name: Supplementary Data 14
Description: Overlap of T2D-associated genes and specific CpG sites from previously conducted prospective or incident T2D studies in blood and differential methylation in the present ***Islet T2D case-control cohort.***

File Name: Supplementary Data 15
Description: Differential gene expression in human islets from non-diabetic donors after silencing by siRNA for *CABLES1, FOXP1, RHOT1,* or *TBC1D4* (five experiments in *n*=5 different donors per group).

File Name: Supplementary Data 16
Description: Transcription factor binding sites in positions -1000 to 0 of RHOT1[39] in regions defined as promoters in human islets based on ATAC-Seq and histone modifications[26], and overlap with differentially expressed genes after silencing of *FOXP1, CABLES1* or *TBC1D4*.

File Name: Supplementary Data 17
Description: Metabolomics of INS-1 β-cells transfected with siRhot1 or siNC (nontemplate control).
