# Supplementary material for: Genes with epigenetic alterations in human pancreatic islets impact mitochondrial function, insulin secretion, and type 2 diabetes
Source: Nat Commun. 2023 Dec 12;14:8040. doi: 10.1038/s41467-023-43719-9 (PMC10716521; doi:10.1038/s41467-023-43719-9)
Supplement: Supplementary file 4 — Supplementary Data 1-17 [file 41467_2023_43719_MOESM4_ESM.zip › Supplementary Data/Supplementary Data 13.docx]

**Supplementary Data 13**. Characteristics of participants in the prospective matched case-control study of EPIC-Potsdam

| **Characteristics** | **Controls** | **Cases** | **P-value** |
| --- | --- | --- | --- |
| **n (male/female)** | 270 (140/130) | 270 (140/130) | n.s. |
| **Age, mean (SD) [years]** | 54.4 (7.5) | 54.42 (7.5) | n.s. |
| **BMI, mean (SD) [kg/m²]** | 26.1 (3.5) | 30.5 (4.8) | <0.0001 |
| **HbA1c, median (IQR) [mg/dl]** | 5.4 (5.1; 5.7) | 6.1 (5.7; 6.7) |  |

SD, standard deviation; IQR, interquartile range; BMI, body mass index; HbA1c, glycated hemoglobin; n.s., non-significant. P-values are based on a two-sample t-test (two-tailed).
