# Supplementary material for: Genes with epigenetic alterations in human pancreatic islets impact mitochondrial function, insulin secretion, and type 2 diabetes
Source: Nat Commun. 2023 Dec 12;14:8040. doi: 10.1038/s41467-023-43719-9 (PMC10716521; doi:10.1038/s41467-023-43719-9)
Supplement: Supplementary file 4 — Supplementary Data 1-17 [file 41467_2023_43719_MOESM4_ESM.zip › Supplementary Data/Supplementary Data 6.docx]

**Supplementary Data 6**. Clinical characteristics of the 83 donors of pancreatic islets used for pyrosequencing replication*.*

| **Characteristics** | **Controls** | **T2D cases** | **P-value** |
| --- | --- | --- | --- |
| n (male/female) | 59 (34/25) | 24 (17/7) |  |
| Age (years) | 62.4 [43-81] | 62.6 [45-81] | 0.92 |
| BMI (kg/m*^2^*) | 26.1 [18-40.1] | 27.6 [21.6-34.9] | 0.16 |
| HbA1c (mmol/mol) | 36.9 [27-41] | 50.3 [39-86] | 8x10^-15^ |
| Islet purity (%) | 83.8 [70-100] | 82.1 [70-100] | 0.41 |

Data is shown as mean [range]. P-values are based on a two-sample t-test (two-tailed).
