# Supplementary material for: Genes with epigenetic alterations in human pancreatic islets impact mitochondrial function, insulin secretion, and type 2 diabetes
Source: Nat Commun. 2023 Dec 12;14:8040. doi: 10.1038/s41467-023-43719-9 (PMC10716521; doi:10.1038/s41467-023-43719-9)
Supplement: Supplementary file 5 — Reporting Summary [file 41467_2023_43719_MOESM5_ESM.pdf]

nature portfolio

Corresponding author(s): Charlotte Ling

Last updated by author(s): Oct 19, 2023

Reporting Summary

Nature Portfolio wishes to improve the reproducibility of the work that we publish. This form provides structure for consistency and transparency in reporting. For further information on Nature Portfolio policies, see our Editorial Policies and the Editorial Policy Checklist.

Statistics

For all statistical analyses, confirm that the following items are present in the figure legend, table legend, main text, or Methods section.

n/a	Confirmed	
☐	☒	The exact sample size (*n*) for each experimental group/condition, given as a discrete number and unit of measurement
☐	☒	A statement on whether measurements were taken from distinct samples or whether the same sample was measured repeatedly
☐	☒	The statistical test(s) used AND whether they are one- or two-sided<br>*Only common tests should be described solely by name; describe more complex techniques in the Methods section.*
☐	☒	A description of all covariates tested
☐	☒	A description of any assumptions or corrections, such as tests of normality and adjustment for multiple comparisons
☐	☒	A full description of the statistical parameters including central tendency (e.g. means) or other basic estimates (e.g. regression coefficient) AND variation (e.g. standard deviation) or associated estimates of uncertainty (e.g. confidence intervals)
☐	☒	For null hypothesis testing, the test statistic (e.g. *F*, *t*, *r*) with confidence intervals, effect sizes, degrees of freedom and *P* value noted<br>*Give P values as exact values whenever suitable.*
☒	☐	For Bayesian analysis, information on the choice of priors and Markov chain Monte Carlo settings
☒	☐	For hierarchical and complex designs, identification of the appropriate level for tests and full reporting of outcomes
☐	☒	Estimates of effect sizes (e.g. Cohen's *d*, Pearson's *r*), indicating how they were calculated

Our web collection on statistics for biologists contains articles on many of the points above.

Software and code

Policy information about availability of computer code

Data collection	GenomeStudio V2011.1
Data analysis	The following packages and software have been used:<br><br>GraphPad Prism v9.0.0<br>Expression Console Software v1.4.1.46<br><br>DNA methylation data from human islets was analyzed using R (programming language, v3.4.3), Bioconductor v3.6, lumi package v2.30, BMIQ v1.4, COMBAT (sva package, v3.20)<br><br>The methylation data from EPIC-Potsdam was analyzed using meffil (PMID: 29931280) to process the idat files, R version 4.3.0, Platform: x86_64-pc-linux-gnu (64-bit), Running under: Ubuntu 18.04.5 LTS, matrix products: default, BLAS: /usr/lib/x86_64-linux-gnu/atlas/libblas.so.3.10.3, LAPACK: /usr/lib/x86_64-linux-gnu/atlas/liblapack.so.3.10.3; LAPACK version 3.7.1<br><br>Code Availability<br>The computer code used to generate the results described in the methods section under DNA methylation analysis and Statistical methods and more details are available from the corresponding author on request.

For manuscripts utilizing custom algorithms or software that are central to the research but not yet described in published literature, software must be made available to editors and reviewers. We strongly encourage code deposition in a community repository (e.g. GitHub). See the Nature Portfolio guidelines for submitting code & software for further information.

Data

Policy information about availability of data

All manuscripts must include a data availability statement. This statement should provide the following information, where applicable:

- Accession codes, unique identifiers, or web links for publicly available datasets
- A description of any restrictions on data availability
- For clinical datasets or third party data, please ensure that the statement adheres to our policy

Data Availability
Source data are provided with this paper. The human islet DNA methylation and RNA-seq datasets generated for this study (EPIC DNA methylation data, accession numbers LUDC2022.05.011, LUDC2022.05.012 and RNA-seq, accession number LUDC2022.05.013) were deposited in the LUDC repository (https://www.ludc.lu.se/resources/repository). Data are available upon request. Individual-level data from the human pancreatic islets are not publicly available due to ethical and legal restrictions related to the Swedish Biobanks in Medical Care Act, the Personal Data Act and European Union's General Data Protection Regulation and Data Protection Act.
Source data underlying relevant panels in Fig. 1-6, and Supplementary Fig. 1-7 are provided with this publication. The following figures have associated raw data based on genome-wide DNA methylation and expression data: Figure 1b-g, Figure 2a-f, Figure 3a-d, Figure 4a-d, Supplementary Figure 1a-m, Supplementary Figure 2a-c, Supplementary Figure 3a-g, Supplementary Figure 4a-g.

Research involving human participants, their data, or biological material

Policy information about studies with human participants or human data. See also policy information about sex, gender (identity/presentation), and sexual orientation and race, ethnicity and racism.

Reporting on sex and gender	The findings of this project is based on inclusion of both sexes, determined by self-reporting and concordant genetic testing. Sex is considered as a co-variate in all analyses of human islets. The ethical permits do not allow sharing individual level data. Regarding the Islet T2D case-control cohort: among 75 controls, 46 are males and 29 females, and among 25 T2D cases, 17 are males and 8 females. Regarding the Islet HbA1c cohort: among 114 individuals, 73 are males and 41 are females. Regarding EPIC-Potsdam: among 270 controls, 140 are males and 130 females, and among 270 cases, 140 are males and 130 females.
Reporting on race, ethnicity, or other socially relevant groupings	All pancreatic islets are from donors from The Nordic Network for Clinical Islet Transplantation Program (www.nordicislets.org) from Scandinavia, a defined geographic region. Clustering of existing GWAS data from this population suggests that a small number of donors are of non-Scandinavian descent (data not shown). EPIC-Potsdam is from the Potsdam region in Germany and there is no reporting on ethnicity of this population. There is no reporting on any other socially relevant groupings.
Population characteristics	Regarding the Islet T2D case-control cohort: 75 controls with an age of 61.4 (43-81) years, BMI of 25.9 (18-40.1) kg/m2, and HbA1c of 36.7 (23-41) mmol/mol, and 25 cases with an age of 62.8 (45-81) years, BMI of 27.4 (21.6-34.9) kg/m2 and HbA1c of 50.1 (39-86) mmol/mol. The ethical permits do not allow sharing individual level clinical data in public domains.<br>The Islet HbA1c cohort include 114 individuals with age of 58.8 (24-81) years, BMI of 26.3 (18-40.1) kg/m2, and HbA1c of 38.8 (23-70) mmol/mol. The ethical permits do not allow sharing individual level data.<br>Regarding EPIC-Potsdam: 270 controls with an age of 54.4 (7.5) years, BMI of 26.1 (3.5) kg/m2 and HbA1c of 5.4 (5.1-5.7) mg/dl and 270 cases with an age of 54.42 (7.5) years, BMI of 30.5 (4.8) kg/m2 and HbA1c of 6.1 (5.7-6.7) mg/dl. The controls and cases have same gender distribution.
Recruitment	Donors of pancreatic islets were from the Scandinavian Transplantation Unit and included multi-organ human donors. Islets were included in this study when not used for transplantation due to clinical reasons. There may be a bias in who is willing to transplant organs for research. However, it is impossible to dissect how such bias would impact our results.<br><br>The recruitment in EPIC-Potsdam was conducted between 1994 and 1998, and the final censoring date was August 2005. Between 1994 and 1998, potential study participants were drawn from the population registers of Potsdam and the surrounding area using a random procedure. The participants were invited to the study center, examined extensively (e.g. measuring blood pressure and determining body dimensions) and asked about their eating habits and lifestyle. Blood samples were also taken, which we keep in our biobank. The initial invitation to EPIC-Potsdam was random and should hence not contribute to any bias. However, it is possible that individuals who participated in the study contribute to a bias. However, it is impossible to dissect if such bias impact our results.
Ethics oversight	All procedures regarding the human pancreatic islets were approved by the Swedish Ethical Review Authority. The EPIC-Potsdam study procedures were approved by the Ethics Committee of the Medical Association of the State of Brandenburg (Germany). Human studies followed the Helsinki Declaration and written informed consent was obtained from pancreatic donors or their relatives and from participants of the EPIC-Potsdam.

Note that full information on the approval of the study protocol must also be provided in the manuscript.

Field-specific reporting

Please select the one below that is the best fit for your research. If you are not sure, read the appropriate sections before making your selection.

☒ Life sciences ☐ Behavioural & social sciences ☐ Ecological, evolutionary & environmental sciences

For a reference copy of the document with all sections, see nature.com/documents/nr-reporting-summary-flat.pdf

Life sciences study design

All studies must disclose on these points even when the disclosure is negative.

Sample size

Sample size was determined based on power calculations. These were made based on our previous data, where we have analyzed DNA methylation and gene expression in human islet and blood samples/cells.

Data exclusions

No data has been excluded.

Replication

For all experiments, we state in the legends how often the experiments were replicated or performed independently.
Moreover, we have used several different methods/models to test the reproducibility of our data. For example, we used both human pancreatic islet samples from people with type 2 diabetes, who show reduced RHOT1 DNA methylation and mRNA expression, and we then studied the impact of reduced RHOT1 expression in both human islets and rat β-cells using siRNA and in a diabetic animal model (GK rats). All these models support a key role for RHOT1-deficiency in mitochondrial dysfunction, resulting in reduced glucose-stimulated insulin secretion linked to type 2 diabetes.
We also used pyrosequencing to technically validate the differential DNA methylation found in islets from individuals with T2D versus controls when using the EPIC array. Here, we could technically replicate the T2D-associated methylation differences for all our analyzed genes. We also used luciferase assay to show that DNA methylation directly alter the transcriptional activity in β-cells. We used several methods to study mitochondrial dysfunction in in Rhot1-deficient β-cells, e.g., seahorse, Western blot, MitoTracker staining, and PercevalHR. All these methods showed similar results. Moreover, while a large proportion of our data are novel, we could also replicate some of our data based on data in published studies.

Randomization

Samples were randomized to chips for DNA methylation and expression arrays. For functional follow-up experiments in cell-lines and animals, randomization could not fully be used due to the need to know which group each sample belongs to. However, for functional assays, cells were seeded and samples harvested and analyzed in random order when possible. Moreover, siNC and siRhot1 treated cells were loaded in every second well on western blot, to avoid experimental bias. In functional experiments, including cell lines and animals, there are no co-variates to control for.

Blinding

During the genome-wide DNA methylation and gene expression experiments the samples were anonymous to the technicians who run the experiments and hence they did not know which group samples belonged to. Moreover for many of our analyses blinding or no blinding is not an issue, as the results are exact readouts done by plate readers, analysers, etc. However, for some functional experiments in cell lines and animals during for example qPCR and Western blot analysis the technician needed to know the order the samples were loaded on plates and gels and then blinding was not possible.

Reporting for specific materials, systems and methods

We require information from authors about some types of materials, experimental systems and methods used in many studies. Here, indicate whether each material, system or method listed is relevant to your study. If you are not sure if a list item applies to your research, read the appropriate section before selecting a response.

Materials & experimental systems

n/a	Involved in the study	
☐	☒	Antibodies
☐	☒	Eukaryotic cell lines
☒	☐	Palaeontology and archaeology
☐	☒	Animals and other organisms
☒	☐	Clinical data
☒	☐	Dual use research of concern
☒	☐	Plants

Methods

n/a	Involved in the study	
☒	☐	ChIP-seq
☐	☒	Flow cytometry
☒	☐	MRI-based neuroimaging

Antibodies

Antibodies used

The following antibodies were used for western blotting: The primary antibodies were citrate synthase (#14309S, clone D7V8B, 1:1000, Cell Signaling Technologies, Danvers, MA, USA), cytochrome C (#4272T; 1:1000, Cell Signaling Technologies), LAMP1 (ab24170, 1:750, abcam, Cambridge, UK), LC3B (NB100-2220, 1:1000, Novus Biologicals, Centennial, CO, USA), OPA1 (#80471, D6U6N, 1:1000, Cell Signaling Technologies), p62 (ab91526, 1:500, abcam), Parkin (ab77924, clone PRK8, 1:750, abcam), PINK1 (ab23707, 1:750, abcam), RHOT1 (ab188029, clone CL1083, 1:500, abcam), and total OXPHOS rodent antibody cocktail including antibodies against NDUFB8, SDHB, UQCRC2, MTCO1, and ATP5A (#ab110413-MS604, clones 20E9DH10C12, 21A11AE7,

	13G12AF12BB11, 1D6E1A8, and 15H4C4, 1:250, abcam). The HRP-conjugated secondary antibodies were goat anti-rabbit (#7074, 1:10000, Cell Signaling Technologies) and goat anti-mouse (1706516, 1:5000, Bio-Rad).
Validation	All antibodies are commercially available and have been characterized by the manufacturers (and in several publications) for their reactivity in the appropriate species and for their compatibility to be used with the respective application. Moreover, all antibodies were validated by respective supplier by use of, e.g., knockout cells. Conditions for blocking and antibody dilutions are stated in the method section. siRNA-silenced samples were used for validation of anti-Rhot1 antibodies (ab188029, abcam, Fig. 5c, Supplementary Fig. 5g-h). Additionally, antibodies were characterized by the expected regulation pattern for example p62 (ab91526, abcam) regulation by amino acid starvation. For Western blot we used molecular weight markers to identify the band(s) that migrated at the expected size of each respective protein analyzed. Validation details are also available on the manufacturers' websites.

Eukaryotic cell lines

Policy information about cell lines and Sex and Gender in Research

Cell line source(s)	We used the rat INS-1 832/13 β-cell developed by Hohmeier, H.E. et al (PMID: 10868964). INS-1 cells were developed from a male rat.
Authentication	It was verified that these cells are β-cells based on their ability to secrete insulin and respond to glucose with increased glucose-stimulated insulin secretion (only β-cells produce and secrete insulin). This cell line was not authenticated using any additional authentication techniques.
Mycoplasma contamination	Cells were tested negative for mycoplasma.
Commonly misidentified lines (See ICLAC register)	No commonly misidentified cell lines were used in the study.

Animals and other research organisms

Policy information about studies involving animals; ARRIVE guidelines recommended for reporting animal research, and Sex and Gender in Research

Laboratory animals	We used male GK rats, developed by selective breeding of Wistar rats (PMID: 32180184) and control Wistar rats from Janvier labs in France. Animals were kept in standard controlled housing conditions; 21–22°C, 55–65% humidity and 12-h light/12-h dark cycle and given standard chow and water ad libitum. The animals were used at 8 or 12 weeks of age.
Wild animals	No wild animals were used in the study.
Reporting on sex	Regarding animals, we only use one sex. The main reason is to reduce the number of animals included in the experiments according to 3R principle (Replacement, Reduction and Refinement). It is previously known that both male and female GK rats have reduced insulin secretion. Moreover, in all our human experiments both sex were included.
Field-collected samples	No field collected samples were used in the study.
Ethics oversight	Animal experiments were performed with permission of the Animal Ethics Committee of Lund University (Permit number 5.8.18-04115/2021) in accordance with the legal requirements of the European Community (86/609/EEC).

Note that full information on the approval of the study protocol must also be provided in the manuscript.

Flow Cytometry

Plots

Confirm that:

- ☒ The axis labels state the marker and fluorochrome used (e.g. CD4-FITC).
- ☒ The axis scales are clearly visible. Include numbers along axes only for bottom left plot of group (a 'group' is an analysis of identical markers).
- ☒ All plots are contour plots with outliers or pseudocolor plots.
- ☒ A numerical value for number of cells or percentage (with statistics) is provided.

Methodology

Sample preparation	Transfected INS-1 823/13 β-cells were washed with 300 µL of pre-warmed PBS + 2% fetal calf serum (FCS) and centrifuged for 5 min at 300g. Cells were resuspended in 50 µL of PBS + 2% FCS and stained with 10 µM MitoSox Red (ThermoFisher Scientific, M36008) for 15 min at 37°C. After staining the β-cells, 300 µL of PBS + 2% FCS was added to wash them. After centrifuging for 5 min at 300g, β-cells were resuspended in PBS + 2% FCS and directly acquired in the flow cytometer (CytoFLEX Beckman Coulter).

Instrument	CytoFLEX Beckman Coulter
Software	FlowJo
Cell population abundance	This is not applicable since these cells were not sorted.
Gating strategy	The gating strategy is displayed in Supplementary Figure 6d.

☒ Tick this box to confirm that a figure exemplifying the gating strategy is provided in the Supplementary Information.
